# An overview of role stress theory and recent research advances in the management of newly qualified nurses: a narrative review

**DOI:** 10.3389/fmed.2026.1851114

**Published:** 2026-07-30

**Authors:** Hailei Bian, Lingjun Li, Qingqing Du, Li Qiao, Li Ni, Wenwen Tong

**Affiliations:** 1 Shanghai East Hospital, School of Medicine, Tongji University, Shanghai, China; 2 Shanghai Children's Hospital, School of Medicine, Shanghai Jiao Tong University, Shanghai, China; 3 Tongji University School of Medicine, Shanghai, China

**Keywords:** emotional intelligence, management strategies, newly qualified nurses, physical and mental health, role stress theory

## Abstract

Originating in sociology, role stress theory is one of the key theories in the fields of organizational behavior and psychology. It primarily examines the stress experienced by individuals in specific roles and its effects. To date, extensive basic and empirical research has been conducted in various fields, including education and healthcare. Newly qualified nurses generally experience high levels of role stress, which causes various discomforts in their work and poses challenges for nursing human resource management. An in-depth exploration of the current characteristics of role stress among newly qualified nurses and corresponding management strategies holds significant theoretical value and practical significance. This narrative review synthesizes existing evidence-based findings. Recommendations for the intervention and management of role stress among newly qualified nurses should adhere to the core principles of early intervention, continuous support, and multidimensional integration. By systematically integrating individual psychological interventions with organizational management interventions and addressing the evolving needs of newly qualified nurses at different stages, it is possible to fundamentally improve their role adaptation and achieve the dual objectives of increasing retention rates and ensuring the quality of nursing care. Future research should focus on enhancing the methodological quality of intervention studies, conducting more high-quality randomized controlled trials, and integrating intelligent management technologies. It should also strengthen the tracking and evaluation of long-term intervention outcomes to continuously optimize management strategies and create a more supportive work environment for the growth and development of newly qualified nurses.

## 1 Introduction

The shortage of nursing human resources is one of the major challenges currently facing global health systems. A 2020 report by the World Health Organization (WHO) indicated that the global nursing shortage stands at 5.9 million, with 89% of this shortage concentrated in low- and middle-income countries. It is projected that by 2030, global health systems will continue to face a severe nursing workforce shortage (1). In China, although the total number of registered nurses has increased from 2.048 million in 2010 to 5.22 million in 2022, the nurse-to-population ratio (approximately 3.7 nurses per 1,000 people) remains below the international average, and the structural shortage of nursing staff remains severe (1, 2).

Newly Qualified Nurses (NQNs) or Newly Graduated Nurses (NGNs) refer to registered nurses who have recently graduated from nursing schools and passed the licensing examination but lack clinical experience (typically defined as having been employed for ≤2 years) (3). As a vital reserve force within the nursing workforce, the retention and stability of NQNs are indispensable for alleviating nursing shortages and ensuring the quality of care. A meta-analysis based on 8,593 nurses across eight countries showed that the incidence of turnover intention among newly qualified nurses ranged from 6 to 61% (4). Meanwhile, a study conducted in Grade A tertiary hospitals nationwide in China revealed that 33.5% of newly qualified nurses expressed a desire to leave, with 3.8% having a very strong intention to resign (5). At the same time, a large body of research indicates that new nurses face significant physical, mental, and emotional challenges, leading to turnover rates significantly higher than those of experienced nurses. High attrition among new nurses not only results in substantial losses in human resources and training costs for healthcare institutions but also has profound negative impacts on nursing team stability and patient safety (6–9). How to help new nurses successfully navigate the first 2 years of their career and stabilize the nursing workforce has become an urgent issue in nursing management. In exploring the underlying causes of new nurse turnover, role stress is considered by the academic community to be one of the most explanatory core variables (10, 11). As new nurses transition from interns to licensed nurses, they must simultaneously assume multiple roles, including clinical caregiver, order taker, patient educator, and team collaborator. However, their lack of practical experience, underdeveloped professional skills, and unclear role definitions make them highly susceptible to self-doubt, anxiety, and a lack of professional identity in complex clinical settings (12).

Role Stress Theory originated in sociology. Since its introduction by Kahn et al. in 1964, it has provided a systematic analytical framework for research in social psychology and organizational behavior (13). The theory categorizes role stress into three types: role ambiguity, role conflict, and role overload. It reveals how role factors in organizational environments influence individuals’ psychological behaviors and work status, and has been the subject of extensive basic and empirical research across various fields, including education and healthcare (13). Among these, nursing-related empirical studies have successively confirmed that high levels of role stress are significantly associated with new nurses’ occupational burnout, anxiety and depression, decreased job satisfaction, and increased turnover intentions, and indirectly threaten patient safety by influencing nurses’ nursing behaviors (14–16). Therefore, using role stress theory as a framework to thoroughly explore the current characteristics of role stress among new nurses and corresponding intervention strategies holds significant theoretical value and practical significance. This review aims to comprehensively summarize research progress on role stress theory in the field of new nurse management. It focuses on a comprehensive analysis across three dimensions: the theoretical implications of role stress theory, the current status of role stress among new nurses, and intervention strategies. The review seeks to provide theoretical references and practical guidance for nursing managers in developing scientifically sound and effective management plans for new nurses, while also aiming to guide future research in this field.

## 2 Methodology

This study adopted a narrative review methodology, conducting a comprehensive but non-systematic search of domestic and international academic literature on role stress theory and its relationship to new nurses’ role adaptation, occupational stress, and transition interventions. The study aimed to summarize the current state of research, application outcomes, and research limitations in this field. Chinese search databases included China National Knowledge Infrastructure (CNKI), Wanfang Database, VIP Chinese Science and Technology Journal Database, and China Biomedical Literature Database (CBM); English search databases included PubMed, Web of Science, Embase, and CINAHL. Given the early origins of role stress theory, the search period was set from January 1960 to December 2025, incorporating retrospective searches for classic foundational literature.

Search terms included: “role stress,” “role conflict,” “role ambiguity,” “role overload,” “newly graduated nurse,” “newly qualified nurses,” “newly licensed nurse,” “novice nurse,” “nursing management,” “transition shock,” “nurse residency,” “nursing turnover,” “occupational stress,” and “nursing management.” A combination of subject headings and free-text terms was used, with Boolean operators employed to construct search queries. Additionally, the reference lists of relevant high-quality reviews were manually traced to identify any omitted literature, ensuring the comprehensiveness of the search.

Inclusion criteria: (1) Publication date: January 1960 to December 2025; (2) Study population: Registered nurses with ≤2 years of experience; (3) Research topic: Variables related to role stress; (4) Document type: Original research, systematic reviews, meta-analyses, and reviews; (5) Language: Chinese or English. Exclusion criteria: (1) Duplicate publications; (2) Abstracts or full texts unavailable; (3) Study subjects who are not nurses.

An initial search using the established strategy yielded 1,589 articles. After importing these into literature management software and removing duplicates, 1,027 articles remained. Through preliminary screening of titles and abstracts, 731 articles were excluded for failing to meet the thematic criteria, target population requirements, or being irrelevant. The remaining 296 articles were reviewed in full, and a secondary screening was conducted strictly according to the inclusion and exclusion criteria. Ultimately, 65 eligible articles were included, forming the analytical basis for this review.

## 3 Overview of role stress theory

### 3.1 Origins and development of role stress

Role Stress Theory was established by Kahn et al. based on the Role Episode Model. Through a large-scale survey of 53 organizations in the United States, they revealed the widespread existence of role conflict and role ambiguity, as well as their negative impact on employees’ mental health and work behavior, thereby laying the conceptual foundation and empirical tradition for research on role stress in organizational contexts (13). Subsequently, Rizzo et al. further developed an operationalized measurement system for role conflict and role ambiguity based on Kahn’s framework; the scale they developed remains one of the most widely cited measurement tools in the field to this day (17).

By the 1980s, the academic community introduced the dimension of role overload to the two-dimensional framework of role conflict and role ambiguity, forming a three-dimensional theoretical structure for role stress research. Beehr et al. defined the conceptual distinction between quantitative role overload (referring to a task load exceeding an individual’s capacity for time and energy) and qualitative role overload (referring to task difficulty exceeding an individual’s capabilities) from the perspective of work demands, further enriching the theoretical implications of role stress (18).

Since Kramer introduced the concept of “reality shock” in Reality Shock: Why Nurses Leave Nursing in 1974, research on the role adaptation of new nurses has gradually evolved from a description of the school-clinic gap to an explanatory framework integrating multiple theories. Kramer’s reality shock theory reveals the feelings of disillusionment, anxiety, and difficulties in role adaptation that new nurses experience upon entering clinical practice due to the discrepancy between educational ideals and workplace realities (19). Benner’s “Novice to Expert” model further explains why new nurses are prone to role overload in complex situations from the perspective of clinical competency development (20). The “Experiencing Transitions” theory proposed by Meleis et al. provides a middle-range theoretical foundation for understanding the shifts in identity, relationships, competencies, and environment as new nurses transition from students to clinical nurses (21). Building on this foundation, Duchscher proposed the Transition Shock Theory. This framework systematically explains the emotional, physical, sociocultural and developmental, and intellectual shocks experienced by new nurses during the early stages of transition and integrates them into a dynamic adaptation model, making it one of the most widely cited theoretical frameworks in current research on new nurses (14).

### 3.2 Definition of core concepts in role stress

#### 3.2.1 Role conflict

Role conflict arises from contradictions or inconsistencies between the multiple role expectations an individual must fulfill. When an individual finds it difficult to simultaneously meet the expectations of different role sources, psychological conflict and tension arise. Role conflict encompasses not only intra-role conflict but also extra-role conflicts such as work–family conflict or gender role conflict. Kahn et al. classify role conflict into the following four types (13): Intrasender Conflict: contradictory role expectations from the same role sender; Intersender Conflict: conflicting role expectations from different role senders; Inter-role Conflict: conflicting expectations among the different social roles an individual plays; Person-Role Conflict: conflict between role expectations and the individual’s own values or capabilities.

Among new nurses, role conflict primarily manifests as discrepancies in expectations between healthcare providers (Intersender Conflict), contradictions between professional demands and personal values (Person-Role Conflict), and conflicts between work and family roles (Inter-role Conflict). These conflicting role expectations may cause new nurses to feel confused and stressed, affecting their job satisfaction and professional identity.

#### 3.2.2 Role ambiguity

Role ambiguity refers to a psychological state in which an individual lacks a clear understanding of their role responsibilities, task boundaries, work objectives, and evaluation criteria; its essence lies in the insufficiency or lack of clarity regarding role-related information (13). Rizzo et al. point out that the core of role ambiguity does not lie in an absolute lack of information, but rather in the individual’s insufficient subjective perception of existing information, a perception influenced by the individual’s level of experience, cognitive style, and the quality of organizational communication (17). Role ambiguity is particularly pronounced among new nurses, manifesting primarily in the following three aspects: unclear understanding of the scope of job responsibilities, insufficient grasp of performance evaluation criteria, and limited knowledge of departmental regulations and work procedures.

#### 3.2.3 Role overload

Role overload refers to a situation where the role expectations placed on an individual exceed the capacity of their available resources in terms of quantity or difficulty. It is divided into two forms: quantitative overload (excessive task volume) and qualitative overload (excessive task difficulty) (18). For new nurses, quantitative overload primarily stems from an excessive workload caused by a low nurse-to-patient ratio, while qualitative overload primarily results from the gap in competence between their limited clinical experience and complex nursing tasks. Research indicates that the mechanisms through which quantitative and qualitative overload affect new nurses differ: quantitative overload primarily triggers emotional exhaustion directly through time pressure, whereas qualitative overload more often influences occupational burnout indirectly through the erosion of self-efficacy (22). These three dimensions are both independent and interrelated, and they frequently coexist and overlap within the same individual, forming a state of complex role stress that produces synergistic negative effects on the physical and mental health of new nurses.

### 3.3 Theoretical models related to the mechanisms of role stress

#### 3.3.1 Job demands-resources model

The Job Demands-Resources Model (JD-R), proposed by Bakker and Demerouti, is one of the most widely cited theoretical frameworks for explaining the relationship between role stress and occupational burnout among nurses (23). This model posits that job characteristics can be divided into two major categories: job demands (e.g., high nurse-to-patient ratios, complex nursing tasks) and job resources (e.g., social support, mentor guidance, job autonomy). Role conflict, role ambiguity, and role overload are all typical job demands. When job demands consistently exceed the compensatory capacity of job resources, individuals initiate a health impairment process, manifesting as emotional exhaustion and occupational burnout; conversely, adequate job resources promote work engagement through a motivational process, thereby buffering the health-damaging effects of stress (23). The core contribution of the JD-R model lies in revealing the interactive effects of job demands and job resources, providing a theoretical foundation for role stress interventions at the organizational level.

#### 3.3.2 Conservation of resources theory

The Conservation of Resources Theory (COR), proposed by Hobfoll, posits that individuals possess an intrinsic motivation to acquire, maintain, and protect valuable resources (including material, social, personal trait, and energy resources) (24). Stress arises when resources face actual loss, the threat of loss, or when expected returns are not achieved after resource investment. For new nurses, the essence of role stress is a continuous process of depleting multiple resources: role conflict depletes emotional resources, role ambiguity depletes cognitive resources, and role overload depletes time and physical resources. When the rate of resource depletion consistently exceeds the rate of resource replenishment, the individual enters a “loss spiral,” becoming trapped in a vicious cycle where coping with stress becomes increasingly difficult (24). The COR theory provides an important explanatory framework for understanding the cumulative effects of role stress and the decision-making process leading to resignation. It is worth noting that the concept of “personal trait resources” in COR theory provides a starting point for understanding the moderating role of individual differences in role stress. Emotional intelligence, as a personal resource that can be cultivated and developed, has received increasing attention in recent years in research on stress among new nurses (25, 26).

#### 3.3.3 Transition shock theory

**Figure 1:**
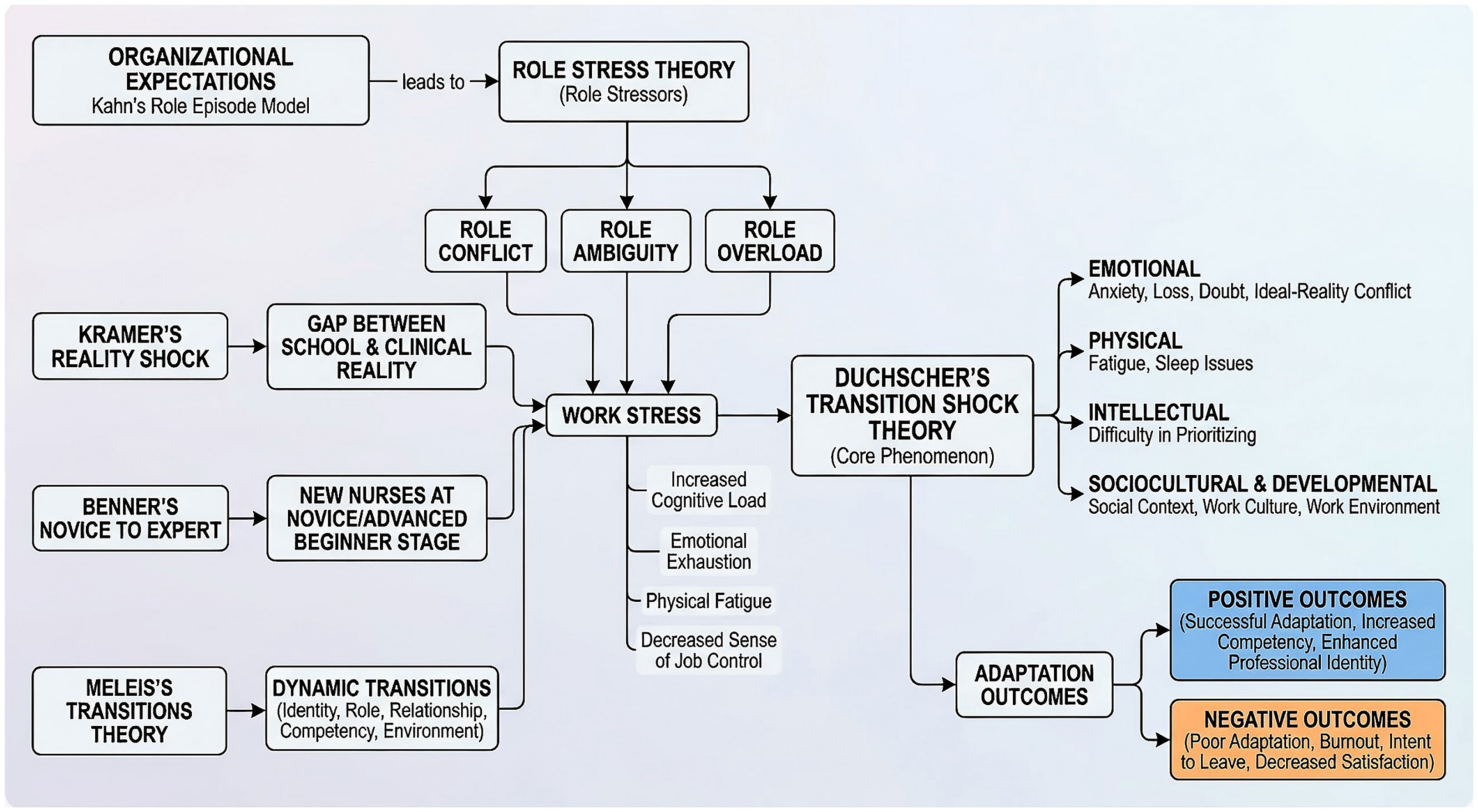
Conceptual diagram of relationships among theoretical models related to the mechanism of role stress.

Transition Shock Theory was proposed by Duchscher specifically to address adaptation issues during the transition period for new nurses and is currently the most targeted theoretical framework in the field of new nurse role stress research (14). This theory posits that the transition of new nurses from the student role to the clinical nurse role is a process accompanied by a strong “transition shock,” the core characteristic of which is that individuals simultaneously face intense transitional stress across four dimensions: emotional, physical, sociocultural and developmental, and intellectual. Duchscher divides the role adaptation process of new nurses into three stages: the Doing stage (0–3 months after employment, characterized by high anxiety and role ambiguity), the Knowing stage (3–5 months after employment, characterized by the awakening of critical awareness and the emergence of role conflict), and the Being stage (5–12 months after employment, characterized by the gradual establishment of professional identity) (14). If new nurses do not receive sufficient support during this transition, they may remain trapped in a state of self-doubt and fear, prolonging the duration of the transition shock and increasing the likelihood of sliding into a transition crisis. The Transition Shock Theory provides an important temporal reference framework for designing phased intervention strategies to address role stress in new nurses. The relationships among theoretical models related to the mechanisms of role stress are illustrated in Figure 1.

**Figure 2:**
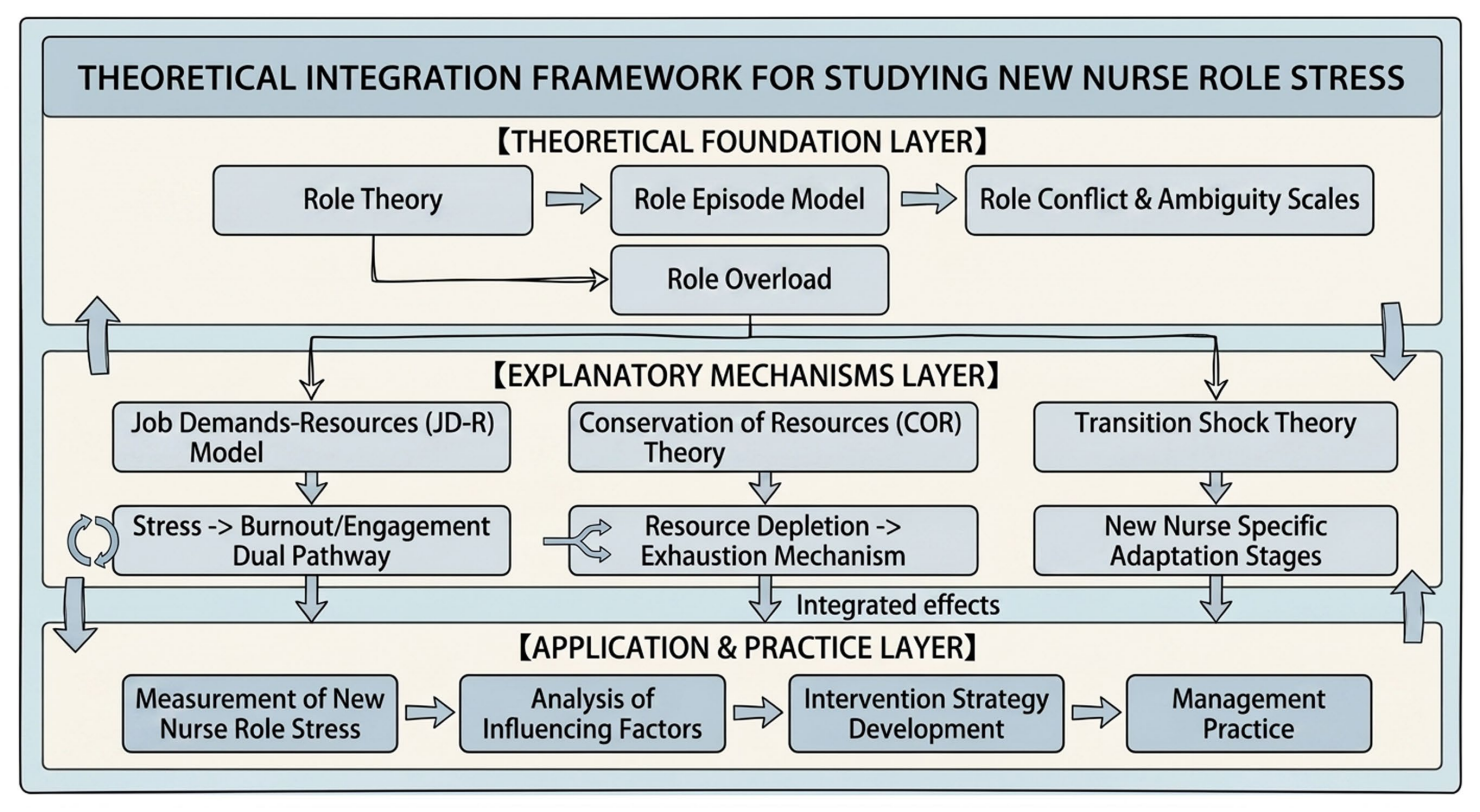
Conceptual diagram of the relationships among theoretical models related to nurse transition.

The developmental trajectory of theoretical models related to the mechanisms of role stress reveals that the theoretical understanding of role stress among new nurses has evolved from a unidimensional conceptual definition (role conflict) to a multidimensional structural framework (the three-dimensional framework of role conflict, role ambiguity, and role overload), and further to an integrated multi-theoretical explanation (the JD-R model, COR theory, and transition shock theory), with the depth of research continuously increasing. These theories build upon one another; the conceptual framework of the relationships among theoretical models related to nurse transition is shown in Figure 2.

However, existing theoretical frameworks primarily stem from the Western tradition of organizational behavior, and domestic research in this area began relatively late. Specifically, theoretical research on role stress during the transition period for new nurses within the context of Chinese medical culture is virtually nonexistent. In light of this, the theoretical framework regarding work-related stress among new nurses requires further refinement and supplementation. In particular, its adaptability to China’s cultural and disciplinary context still needs systematic validation. Constructing an integrated theoretical framework based on localized dimensions, such as “face” pressure, role expectations within mentor-apprentice relationships, and role overload under performance evaluations, could serve as a key direction for future theoretical research in this field.

### 3.4 Scales for new nurses based on role stress theory

In terms of measurement tools, Kramer et al. used the Environmental Reality Shock-Related Issues and Concerns (ERS-RIC) scale in their study of a residency program for new nurses to assess the reality shock issues faced by newly licensed nurses during their professional socialization process (27). Kim et al. developed the Transition Shock Scale for Newly Graduated Nurses (TSS-NGN) based on a sample of newly graduated nurses in South Korea. This scale measures the emotional, physical, interpersonal, role responsibility, knowledge and skill, and work environment-related shocks experienced by new nurses during their transition from the student role to the clinical nurse role (28). In terms of domestic research, the Transition Shock Evaluation Scale developed by Xue et al. in 2015 sparked a surge of interest in the transition shock experienced by new nurses in China and provided a localized measurement tool for subsequent studies (29). A comparison of the three scales is presented in Table 1. Overall, the ERS-RIC places greater emphasis on the gap between expectations and reality, the TSS-NGN highlights the multidimensional mechanisms of transition shock, while the Chinese scale is more suitable for screening and evaluation in domestic nursing management practice.

**Table 1:** Comparison of dimensions across measurement scales of the new graduate nurse role stress theory.

Comparison dimensions	ERS-RIC	TSS-NGN	Xue Youru transition stress evaluation scale
Developer/Year	Kramer et al. (2013) (27)	Kim et al. (2017) (28)	Xue et al. (2015) (29)
Dimensions	No clear, stable sub-dimensions have been established.	Six dimensions: (1) conflict between theory and practice; (2) excessive workload; (3) loss of social support; (4) withdrawal from colleague relationships; (5) confusion regarding nursing professional values; (6) work-life imbalance.	Four dimensions: (1) Physical; (2) Psychological; (3) Knowledge and Skills; (4) Sociocultural and Developmental.
Reliability	Statistical reliability and validity have not been fully verified, and sub-domains are not clearly presented.	Total scale Cronbach’s *α* = 0.89; Cronbach’s *α* for each dimension = 0.68–0.81.	Total scale Cronbach’s α = 0.918; Cronbach’s α for each dimension = 0.864–0.940; MIIC = 0.293.
Validity	Content validity confirmed through expert consultation; no independent confirmatory factor analysis report available for structural validity.	CVI = 0.91; EFA identified 6 factors, explaining 71.26% of total variance; CFA model fit was good, RMSEA = 0.05.	CVI = 0.906; EFA identified 4 factors, accounting for 68.538% of the total variance; CFA model fit was good, RMSEA = 0.061.
Target population	Newly licensed nurses participating in the U.S. Nurse Residency Program (NRP).	Newly graduated nurses in South Korea (within 12 months of employment).	Newly hired nurses in China (within 12 months of employment).
Advantages	Long-established theoretical foundation; suitable for assessing the relationship between the work environment and the reality of the job; applicable to nurse residency programs and healthy work environment research.	Specifically developed to address the transition shock experienced by new graduate nurses; clear dimensions; few items, low burden; simultaneous completion of EFA and CFA; clear relationship with burnout and intention to leave.	China-specific tool; large sample size; comprehensive dimensional coverage; high reliability; suitable for screening and management evaluation of transition stress among new nurses in China.
Disadvantages	Relatively insufficient psychometric evidence; lacks clear sub-dimensions; requires caution for cross-cultural use; focuses more on “environmental reality shock” rather than the complete “transition shock”.	Developed within a South Korean cultural context; cross-cultural application requires validation; some dimensions have few items; the “Theory-Practice Conflict” dimension has an α of 0.68, slightly below common standards.	Dimensions are relatively broad and do not allow for the same level of granularity as the TSS-NGN in distinguishing work, relationship, value, and life conflicts; the criterion measure uses self-report rather than an established gold standard; requires further validation across different regions and hospital tiers.

## 4 Current status and characteristics of role stress among new nurses

### 4.1 Overall level of role stress among new nurses

#### 4.1.1 New nurses experience high levels of role stress

Casey et al. found that newly graduated nurses generally experience high levels of work-related stress during the initial phase of employment. Major sources of stress include insufficient clinical competence, lack of confidence in handling emergencies, excessive workload, difficulties communicating with physicians and senior nurses, fear of making mistakes, and difficulty completing the role transition from student to independent practitioner (30). A U.S. study surveyed 402 new nurses and found that their scores on the New Nurse Work Stress Scale averaged 2.07, indicating moderate work stress. High patient demands, difficulties operating medical equipment, and insufficient clinical skills were identified as the primary sources of work stress they faced (31).

Compared to experienced nurses, new nurses typically experience higher levels of role-related stress. In their study on occupational stress among nurses, Chang et al. noted that nursing stress is closely related to clinical experience, job adaptation, and a sense of control over work. Nurses with insufficient clinical experience are more prone to high stress due to demanding work requirements, limited decision-making autonomy, and scarce coping resources (32). This is because experienced nurses have developed a relatively stable professional role identity, clinical judgment, crisis management skills, and team communication patterns through long-term clinical practice, whereas new nurses are still in the role-learning and role-construction stages. For new nurses, due to insufficient clinical experience, low task proficiency, and time management skills still in development, the same workload is often experienced as a higher level of role-related stress. This excessive work pressure may not only lead to physical fatigue but also trigger psychological stress, affecting new nurses’ job performance and physical and mental health.

#### 4.1.2 Role overload is the primary stressor faced by new nurses across countries

An integrative review by Labrague and McEnroe-Petitte of 17 studies found that role overload and role ambiguity were the two most frequently cited sources of stress among new nurses during their first year of employment, and were mentioned by respondents in all included studies (33). A meta-analysis by Yang et al., incorporating 12 studies, confirmed that not only is role stress positively correlated with turnover, but an analysis of its specific dimensions also revealed that role conflict, role ambiguity, and role overload are all positively associated with nurses’ intention to leave, with this association being particularly strong among new nurses within their first 2 years of employment (15). A qualitative study by Fink et al. on the outcomes of residency-style training programs for new nurses also revealed that the reality shock experienced by new nurses largely stems from the intensity of clinical work, increased responsibilities, and demands for independent practice. The enormous workload during the first year of employment can easily overwhelm new nurses, and these factors are intrinsically closely related to role overload (34, 35).

There are three primary reasons why new nurses are more susceptible to role overload. (1) New nurses lack sufficient proficiency in clinical skills and workflows, often requiring more time and cognitive resources to complete the same nursing tasks. (2) New nurses have limited ability to anticipate changes in patients’ conditions and unexpected events, making them more prone to feelings of anxiety and uncertainty when facing complex clinical situations. (3) New nurses typically occupy lower positions in the organizational hierarchy, with limited autonomy and ability to mobilize resources; when staffing is inadequate or team support is insufficient, they are more likely to experience a state of stress characterized by high demands, low control, and low support. Therefore, role overload can be regarded as the most universal and fundamental dimension of role stress among new nurses.

### 4.2 Phases of role stress

The role stress experienced by new nurses exhibits a distinct dynamic evolution over time. Based on the stages outlined in Duchscher’s Transition Shock Theory (14) and in conjunction with existing longitudinal research evidence, this evolution can be summarized into the following three phases:

#### 4.2.1 The initial onboarding phase (0–3 months): a phase of continuously rising stress

New nurses must complete the transition from student to practitioner within a short period while coping with unfamiliar clinical environments and highly uncertain operational situations; role ambiguity and role overload simultaneously reach high levels (14). Hofler and Thomas point out that during the first 3 months of employment, new nurses must simultaneously manage inconsistent expectations from multiple role-senders, including head nurses, physicians, and patients, and the incidence of role conflict is significantly higher than in subsequent stages (36). Taiwan scholar Ye conducted a three-month study of new nurses and found that the intention to leave among new nurses reached as high as 31.5% during the first 3 months (37). If adequate organizational support is not provided during this phase, it is highly likely to induce professional burnout, thereby affecting subsequent career stability (38, 39).

#### 4.2.2 The mid-adaptation phase (3–12 months): a phase of fluctuating stress

As clinical experience gradually accumulates, role ambiguity generally diminishes, and new nurses begin to form their own clinical judgments; however, the likelihood of disagreements with physicians or head nurses increases, leading to intensified role conflict (40). A domestic longitudinal study indicates that role stress among new nurses peaks 4 months after starting work and gradually decreases at 8 and 12 months (41). A one-year longitudinal study by Taiwan scholars Cheng et al. showed that work-related stress among new nurses peaked at 3 months and gradually decreased at 6 and 12 months (42). In some hospitals, the reduction of mentor support after new nurses complete pre-service training can result in a sudden withdrawal of resources, triggering a temporary rebound in role-related stress (43).

#### 4.2.3 The later onboarding phase (1–2 years): a phase of stable stress

Overall, role stress shows a downward trend, while professional identity and self-efficacy gradually increase. However, Bratt and Felzer note that approximately 30% of new nurses still report persistently high levels of role conflict; in the absence of sustained organizational support, role stress in some individuals may evolve into chronic occupational stress (44). A qualitative study by Hayward et al. also found that the source of stress in this phase has shifted from the early-stage “not knowing what to do (role ambiguity)” to “disagreeing with what is required of them (role conflict),” indicating a qualitative transformation in the nature of stress (45). A three-year longitudinal study by Rudman et al. of 1,155 new nurses in Sweden found that work-related stress remained relatively stable during this period, with stress levels peaking in the second year (39).

Understanding the actual levels and dynamic evolution of role stress among new nurses is a crucial prerequisite for developing targeted management intervention strategies. Nursing managers should focus on the prominent issues and influencing factors of work-related stress at different stages of a new nurse’s career. By following the patterns of role stress evolution and competency progression, they should develop stress management strategies tailored to specific timeframes and individuals to help new nurses complete their role transition and adaptation process as smoothly and quickly as possible.

## 5 Management interventions for role stress

### 5.1 Structured pre-employment training

A Structured Orientation Program is a key intervention for alleviating role ambiguity among new nurses during the initial onboarding phase. According to role theory, role ambiguity primarily stems from an individual’s lack of clear understanding of job responsibilities, boundaries of authority and accountability, behavioral standards, and organizational expectations (17). High-quality pre-service training, through systematic introductions to departmental responsibilities, regulations, clinical protocols, healthcare communication processes, and support pathways, helps new nurses more quickly understand organizational rules and role boundaries, thereby reducing uncertainty during the initial onboarding period. The theory of organizational socialization also indicates that a structured organizational integration process can help new nurses learn organizational norms, role expectations, and professional behaviors (46). An integrative review indicates that new nurse transition programs typically include core elements such as structured education, clinical feedback, and socialization support; these measures help improve new nurses’ clinical competence, confidence, job satisfaction, and retention (47). Structured interventions targeting the transition from student to new nurse, such as orientation training and transition practice programs, can improve new nurses’ transition experience and clinical adaptation (48).

Regarding training content, in addition to traditional procedural skills training, we recommend incorporating role expectation management and organizational socialization into the core modules of pre-service training. Upon entering clinical practice after graduation, new nurses often experience a gap between their expectations and reality, including increased responsibilities, a faster work pace, more complex communication, and unclear professional role boundaries. These factors can easily lead to transition shock and role conflict (14, 30). Therefore, pre-service training should help new nurses develop a more realistic understanding of their roles and coping strategies through structured career expectation alignment, job descriptions, real-case discussions, and clarification of role boundaries. This is a crucial pathway to improving their transition experience (46).

In terms of training methods, scenario simulation and role-playing are key tools for enhancing the effectiveness of pre-service training. Simulation training can recreate real clinical stress scenarios in a safe environment, allowing new nurses to practice clinical judgment, communication and coordination, prioritization, and emergency response before they begin working independently. This helps build confidence and reduces feelings of uncertainty in real clinical settings (49, 50). A Chinese study indicates that the scripted role-play model allows new nurses to enter the patient’s perspective through role-swapping, and empathize with patients’ needs. Meanwhile, on-site feedback from trainers and teaching team leaders can promote conscious learning and training in communication, health education skills, interprofessional collaboration, and conflict resolution. This approach helps stimulate new nurses’ interest in training and their proactive learning, improves their professional competence, and serves as an effective training method to facilitate their smooth transition into practice (51).

### 5.2 Nurse residency program (NRP)

Nurse residency programs are key interventions in countries and regions such as the United States and Canada to address the challenges new nurses face in adapting to their roles. These programs typically last 6–12 months, with core components including structured educational curricula, clinical preceptorship or mentorship, reflective sessions, institutionalized feedback, professional socialization support, and career development guidance (47, 52). Among these, preceptorship is the intervention with the strongest evidence within NRPs, identified by multiple systematic reviews and meta-analyses as a key strategy for promoting new nurses’ role adaptation, professional socialization, and retention (47, 48, 53). The core functions of a high-quality preceptorship lie in: helping new nurses enhance clinical competence and reduce role overload through continuous skill demonstration and immediate feedback; reducing role ambiguity by clarifying job responsibilities, workflows, and decision-making boundaries; and mitigating emotional exhaustion caused by role conflict and the transition shock by providing emotional support, outlets for stress relief, and channels for discussing clinical issues (14, 30).

Regarding mentor selection and training, research generally emphasizes that mentors should possess sufficient clinical experience, strong communication and feedback skills, and a willingness to mentor, and should undergo systematic training in mentoring skills to ensure the quality of the mentoring program’s implementation (54, 55). Systematic reviews indicate that mentoring programs have a positive impact on new nurses’ clinical competence, professional socialization, job satisfaction, and retention; however, these effects are influenced by the quality of mentor support, the mentor-mentee relationship, and the availability of organizational resources (53, 56). When mentors have excessive workloads or lack motivation to mentor, the quality of mentoring may decline, potentially leading new nurses to feel unsupported and neglected. Therefore, establishing systems for mentor training, workload compensation, incentive mechanisms, and mentoring quality evaluation is a critical institutional prerequisite for ensuring the sustained and effective operation of the mentoring system.

### 5.3 Standardized training for new nurses in China

Currently, standardized training for new nurses in China remains in an exploratory phase. The “Training Syllabus for Newly Qualified Nurses” issued by the National Health Commission of the People’s Republic of China in 2016 provides a policy framework for institutional interventions addressing role stress among new nurses in China (3). Through systematic rotations, practical skills assessments, and clinical mentoring, standardized training helps new nurses quickly clarify the boundaries of their roles and responsibilities across departments during the early stages of employment, effectively reducing role ambiguity. Research indicates that China’s standardized training program has yielded positive results since its implementation, with new nurses demonstrating improvements in theoretical knowledge, practical skills, and night-shift competency assessments, leading to a significant enhancement in clinical competence (57). At the same time, the training models actively implemented and continuously innovated by most existing tertiary hospitals in China have become a major highlight, such as the Competency Model, the “Nightingale+” model, and the “Sandwich” teaching method. However, since these explorations are conducted as single-center studies within the scholars’ respective hospitals, their generalizability and applicability still require further investigation and research (58–60). Furthermore, most new nurse training programs are designed by the employing hospitals based on their own institutional circumstances, resulting in significant variations in training processes and assessment methods. There is a lack of a unified, scientific, and objective assessment system, as well as an effective training quality supervision system. Consequently, the standardization of standardized training quality for new nurses in China still needs to be strengthened. A detailed comparison between the Nurse Residency Program and China’s standardized training for new nurses is presented in Table 2.

**Table 2:** Comparison of the advantages and disadvantages of the two training programs.

Program name	Core features	Advantages	Disadvantages
Standardized Training Program for New Nurses in China	Based on national policy guidelines, this program emphasizes a two-year standardized training program, departmental rotations, and training in basic theory and skills.	Driven by national-level policies, this approach facilitates widespread and large-scale implementation.	Lack of standardization in training quality, instructor standards, and evaluation systems; relatively weak psychological support, professional identity, and retention interventions.
NRP	Centered on hospitals and specialized programs, with representative initiatives including Vizient/AACN NRP and Versant NRP, these programs emphasize a 6- to 12-month structured transition.	The programs are mature, with comprehensive evaluation systems, and prioritize professional socialization, retention rates, and cost-effectiveness.	Program costs are high, requiring significant resources and organizational support, and there is a lack of nationally unified mandatory standards.

### 5.4 Optimizing the work environment

Appropriate allocation of nursing staff and workload management are critical organizational interventions for alleviating role overload among new nurses. In the management of new nurses, nursing managers should, based on their clinical competency levels, appropriately reduce the number of patients assigned to them during the initial onboarding period and gradually increase their independent care responsibilities as their knowledge, skills, clinical judgment, and ability to prioritize improve, thereby preventing premature role overload from impairing professional adaptation (27, 47). The rational design of flexible scheduling systems also helps alleviate role-related stress for new nurses. It is recommended to minimize consecutive night shifts and high-intensity shifts during the initial onboarding period and, whenever possible, ensure that new nurses work alongside mentors or preceptors to enhance access to real-time guidance, feedback, and clinical support (48, 52).

### 5.5 Development of psychological support systems

Mindfulness-Based Stress Reduction (MBSR) is an important psychological intervention for stress management among nurses and nursing students that has gained prominence in recent years. Previous studies have shown that mindfulness interventions can improve nurses’ stress levels, burnout, emotional regulation, and mental health (61, 62). Among nursing students, MBSR has also been shown to reduce levels of depression, anxiety, and stress while increasing mindfulness (63). By cultivating non-judgmental awareness of present-moment experiences, MBSR helps new nurses alter their automatic response patterns to stressful events, thereby reducing anxiety, depression, and stress reactions (64). Therefore, incorporating mindfulness training into pre-service or early-career psychological preparation programs for new nurses can help enhance their ability to cope with role ambiguity, role conflict, and clinical stress situations.

Employee Assistance Programs (EAPs) provide new nurses with professional, confidential, and accessible psychological counseling and stress management services, serving as a crucial institutional arrangement for systematic mental health support at the organizational level. EAPs typically include one-on-one counseling, crisis intervention, stress management, referral services, group workshops, and online psychological support. Their core characteristics lie in confidentiality, accessibility, and organizational support (65). Establishing an EAP or similar psychological support channels for new nurses in hospital settings helps provide institutionalized resources to support their coping with stress during the role transition period. Similarly, a qualitative study in China indicated that implementing a peer support model during the standardized training period for new nurses can effectively reduce the level of transition shock, lower perceived stress, enhance positive coping abilities, and promote role adaptation and professional growth among new nurses (66).

Balint groups have also garnered significant attention in recent years as a method of psychological support. Centered on case discussions, these groups utilize role-playing and group reflection to help participants understand the inner needs of all parties involved in a conflict, confront and release emotions, and thereby reduce occupational burnout (67). By immersing themselves in role-playing to experience the perspectives of patients, family members, nurses, and other stakeholders, participants demonstrated a significant improvement in their average mood index scores (68). Domestic researchers who combined the CDIO teaching model with Balint group interventions found that new nurses’ core competencies and professional identity were enhanced, and their satisfaction with the training methods and quality was also higher (69). Through role-playing, Balint groups trained nurses to seek understanding and balance stress from multiple perspectives, which aligns closely with the logic of role stress theory.

Developing emotional intelligence is another key focus in building a psychological support system for new nurses. Emotional intelligence refers to an individual’s ability to perceive, evaluate, understand, and regulate their own and others’ emotions; it serves as a vital personal resource for new nurses in coping with role stress and maintaining their physical and mental well-being. A survey study by Mazzella Ebstein et al. of newly hired oncology nurses at a U.S. cancer center showed that new nurses’ emotional intelligence levels were significantly correlated with their choice of coping strategies. New nurses with higher emotional intelligence were more likely to adopt problem focused coping strategies, and reported relatively lower levels of perceived occupational stress, suggesting that emotional intelligence plays an important protective role in the cognitive evaluation of and coping with occupational stress (26). A cross-sectional study by Görgens-Ekermans and Brand among nurses further confirmed that emotional intelligence has a significant moderating effect on the relationship between occupational stress and occupational burnout; nurses with high emotional intelligence exhibited lower levels of emotional exhaustion and better health status when exposed to the same level of stress (25). From a theoretical perspective, emotional intelligence can be integrated into the “personal trait resources” category of the COR theory. By optimizing the cognitive evaluation of stress, promoting emotional regulation, and expanding interpersonal support networks, it can buffer the detrimental effects of role conflict, role ambiguity, and role overload on the physical and mental health of new nurses. Given that emotional intelligence is malleable and trainable, it is recommended that nursing administrators systematically incorporate modules such as emotional awareness training, emotional regulation techniques, empathetic communication, and interpersonal conflict management into the standardized training and psychological support systems for new nurses. These should complement interventions such as mindfulness-based stress reduction and Balint groups to collectively enhance new nurses’ emotional management abilities and role adaptation levels.

### 5.6 Comprehensive evaluation of intervention effects

#### 5.6.1 Quality of evidence in existing intervention studies

Overall, the evidence grade for existing studies on role stress interventions for new nurses remains relatively low. The number of randomized controlled trials (RCTs) is limited; most intervention studies employ single-group pre-post designs or quasi-experimental designs, lacking strict control conditions and blinded designs, and thus carry risks of selection bias and measurement bias. Furthermore, the follow-up periods in existing intervention studies are generally short (mostly within 3–6 months), making it difficult to assess the long-term sustainability of intervention effects (70).

#### 5.6.2 Optimizing the timing and duration of interventions

Existing research generally supports the principle that “early intervention is superior to delayed intervention,” meaning that initiating intervention before the peak of role stress yields better outcomes than waiting until problems manifest before intervening (11). Regarding intervention duration, comprehensive intervention programs lasting ≥6 months (such as the NRP) are significantly more effective than short-term, single-intervention approaches (such as one-time training), suggesting that the continuity and diversity of interventions are key design elements for ensuring effectiveness (71).

### 5.7 Framework for integrating intervention strategies

**Figure 3:**
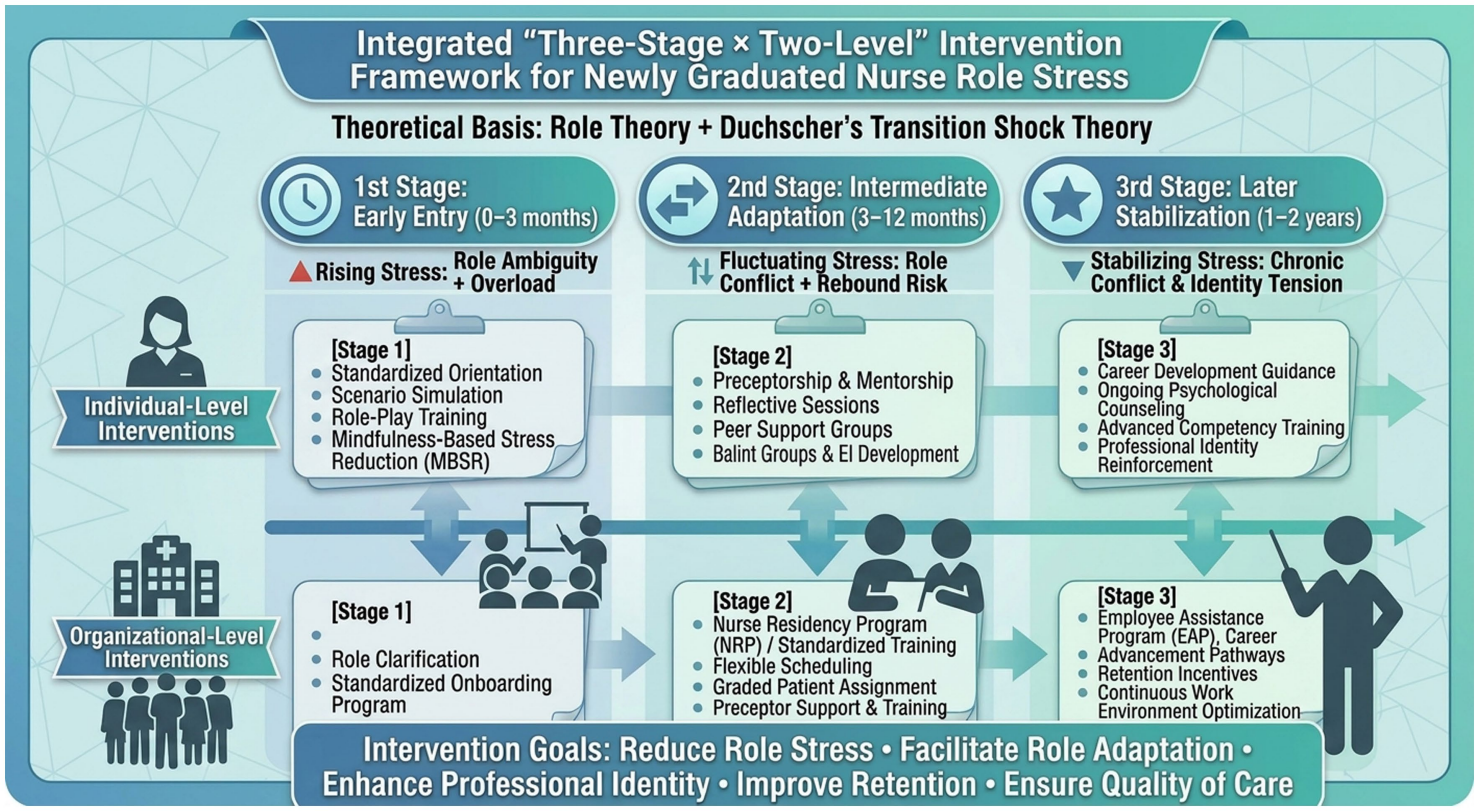
“Three-stage, Two-level” integrated framework for role stress intervention in new nurses.

Based on the current body of evidence, strategies for managing role stress among new nurses should adhere to the core principles of early intervention, continuous support, and multidimensional integration. While single interventions may yield results in specific dimensions, only by systematically integrating individual psychological interventions with organizational control measures, and organically linking pre-employment preparation, adaptation-phase support, and post-employment retention incentives, can we fundamentally improve new nurses’ role adaptation and achieve the dual management goals of reducing turnover rates and ensuring nursing quality. Therefore, this review proposes the following “three-stage, two-level” integrated framework for intervening in new nurses’ role stress, as shown in Figure 3. By providing new nurses with comprehensive “psychological-skills-career planning” support throughout their entire career trajectory, this framework helps them achieve a smooth transition into their professional roles.

## 6 Research gaps and future prospects of role stress theory in the management of new nurses

### 6.1 Research gaps

Although role stress theory has yielded substantial findings in the field of new nurse management, the following significant gaps remain.

#### 6.1.1 Research design: predominantly cross-sectional, lacking longitudinal tracking

The vast majority of existing studies are cross-sectional surveys, which can only describe the level of role stress and its associated factors at a specific point in time. They fail to reveal the dynamic trajectory of role stress during the transition period of new nurses, key turning points, and causal temporal relationships. To date, there is a lack of longitudinal cohort studies in China tracking new nurses from pre-employment through the first 1–2 years of employment, making it difficult to guide precise, phased interventions.

#### 6.1.2 Theoretical level: delayed development of localized theories

Western scholars have developed more than a dozen relevant theoretical models, whereas domestic research remains largely in the preliminary stage of “tool introduction-status quo investigation-factor analysis,” lacking localized theoretical models grounded in the Chinese healthcare cultural context (such as hierarchical concepts, collectivism, and nurse–patient trust relationships). Although existing scales have been translated or adapted, it remains to be verified whether their dimensional structures and item meanings are fully applicable to new nurses in China.

#### 6.1.3 Intervention research: limited methodological diversity and lack of systematic approaches

Current management strategies primarily consist of single measures (such as mentorship programs or psychological training), lacking integrated intervention plans designed to address individual, interpersonal, and organizational levels in a coordinated manner. Most intervention studies lack a randomized controlled trial (RCT) design, feature small sample sizes, and have short follow-up periods, resulting in weak evidence regarding the generalizability and long-term sustainability of intervention effects.

#### 6.1.4 Research population: narrow coverage

Existing research has been overly focused on new nurses in Grade A tertiary hospitals, with very little attention paid to new nurses in settings such as secondary and lower-level hospitals, private hospitals, community health service centers, and long-term care facilities. Additionally, the role stress characteristics of specific subgroups—such as male nurses, nurses from ethnic minority groups, and part-time or contract-based new nurses—have been scarcely explored.

#### 6.1.5 Technology application level: lack of innovative approaches

With the rapid development of artificial intelligence, wearable devices, and mobile health (mHealth), real-time stress monitoring and digital interventions have been implemented in other fields. However, the management of role-related stress among new nurses remains dominated by traditional methods, lacking new assessment and intervention tools based on information technology.

### 6.2 Future prospects

Specific optimization strategies are presented in Table 3.

**Table 3:** Optimization strategies for role stress theory in the management of new nurses.

Optimization dimensions	Optimization strategy	Specific recommendations	Expected outcomes
Research design and methodology	Implementation longitudinal design.	Systematic measurement points will be established at 0, 3, 6, 12, and 24 months after employment; latent variable growth curve models will be used to identify the trajectory of stress evolution.	To clarify the dynamic patterns of role-related stress and establish causal relationships.
	Introduction of a hierarchical linear model.	Employ a Hierarchical Linear Model (HLM) to simultaneously incorporate variables at the individual level (psychological resilience, self-efficacy) and the organizational level (nurse-to-patient ratio, department culture).	Systematically reveal the independent effects and interaction effects of factors at different levels.
	Deepening mixed-methods research.	Organically integrating large-scale questionnaire surveys with in-depth interviews and focus groups, prioritizing an explanatory sequence design.	Achieving a comprehensive understanding of the quantitative patterns and subjective experiences of role stress.
	Applying objective measurement techniques.	Combining Ecological Momentary Assessment (EMA) with wearable devices (HRV, skin conductance, etc.) to establish a dual-track measurement system integrating subjective perception and objective physiology.	Overcome recall bias and enhance the ecological validity of research conclusions.
Theoretical framework	Developing a localized theoretical model.	Based on in-depth qualitative research, construct a localized theoretical framework that integrates the organizational structure of Chinese hospitals, collectivist culture, and power dynamics between medical staff and patients.	Enhancing the theory’s explanatory power in the Chinese context and moving beyond the direct transplantation of Western theories.
	Developing a localized assessment scale.	Develop and revise scales based on the localized theoretical model to establish a nationally standardized assessment scale.	Conducting multi-center, large-scale validation of the assessment scales.
	Incorporate a positive psychology perspective.	Examine the mechanisms by which positive resources—such as psychological capital, professional resilience, and a sense of work meaning—facilitate the professional growth of new nurses, and explore post-stress growth pathways.	Mitigate biases in negative research and provide a positive theoretical foundation for intervention studies.
Intervention studies	Improving the quality of research methodology.	Strictly adhere to the CONSORT guidelines to ensure concealed randomization, assessor blinding, and intention-to-treat (ITT) analysis.	Elevate the level of evidence in intervention studies and enhance the credibility of conclusions.
	Extend the follow-up period for interventions.	Extend the follow-up period from the current 6 months or less to 12–24 months to systematically track the sustained effects of the intervention on role stress, occupational burnout, and turnover behavior.	Assess the long-term sustainability of intervention effects and identify key points where effects may diminish.
	Incorporate health economic evaluations.	Introduce cost-effectiveness analysis (CEA) and cost-utility analysis (CUA) to quantify the input–output ratios of different intervention strategies.	Provide an economic basis for managers to select intervention strategies under resource constraints.
	Explore digital intervention methods.	Systematically evaluate the effectiveness of digital interventions, such as mobile app-based mindfulness training and AI-assisted role-playing simulations of conflict scenarios.	Overcome the time and manpower constraints of traditional interventions to enhance accessibility and personalization.

## 7 Research summary and limitations

### 7.1 Summary

Centered on the core issue of role stress among new nurses, this study conducted a narrative review and comprehensive analysis across three dimensions: theoretical implications, current characteristics, and intervention strategies. A three-dimensional framework comprising role conflict, role ambiguity, and role overload, combined with the JD-R model, COR theory, and the transition shock theory, established a multi-level theoretical system to explain the role stress experienced by new nurses. Existing evidence indicates that new nurses experience significantly higher levels of role stress than experienced nurses, exhibiting a dynamic evolution characterized by a sharp rise in role ambiguity and overload during the initial onboarding phase, the prominence of role conflict during the adaptation phase, and gradual stabilization in the later stages. Regarding role stress intervention strategies, the “three-stage, two-level” integrated framework proposed in this study encompasses measures such as pre-service training and psychological preparation, mentorship programs and standardized training initiatives, as well as workplace optimization and the development of psychological support systems. Evidence-based findings support intervention principles centered on early intervention, continuous support, and multidimensional integration. However, this field still requires further development in terms of constructing localized theories, designing longitudinal studies, and validating high-quality interventions. Future efforts should focus on advancing multicenter longitudinal studies, developing localized tools, and integrating intelligent management technologies.

In summary, the role stress theory provides a theoretical framework for the management of new nurses that is both explanatory and instructive. Against the background of a severe global nursing shortage, establishing a scientific, systematic, and localized role stress management system for new nurses guided by role stress theory holds significant theoretical value and far-reaching practical implications for stabilizing the nursing team, enhancing nursing quality, and ensuring patient safety.

### 7.2 Limitations

This review employed a narrative review methodology and did not conduct systematic literature quality assessment or quantitative meta-analysis, limiting the objectivity and reproducibility of the conclusions. The search was restricted to Chinese and English, potentially omitting relevant studies in other languages, posing risks of language bias and publication bias. The included studies exhibited significant heterogeneity in study design, sample size, and measurement tools. Some conclusions were based on cross-sectional or small-sample studies, resulting in a low level of evidence. Furthermore, the included studies were primarily from Western countries and Class A Grade three hospitals in China, with insufficient coverage of primary care institutions and special populations, limiting the generalizability of the conclusions. Future research should focus on conducting systematic reviews and meta-analyses, and generating higher-level evidence through multicenter randomized controlled trials to provide a more robust foundation for clinical practice guidelines and policy development regarding role stress management for new nurses.
